# Optimizing Infection Risk in Primary Total Knee Arthroplasty: A Narrative Review of Current Concepts, Controversies, and Evidence Gaps

**DOI:** 10.7759/cureus.114886

**Published:** 2026-08-20

**Authors:** Mathias S Salazar, Lisbeth T Almeida

**Affiliations:** 1 Orthopedics and Traumatology, Novaclinica Santa Cecilia, Quito, ECU; 2 Orthopedics and Traumatology, Pontificia Universidad Católica del Ecuador, Quito, ECU; 3 Orthopedics and Traumatology, Hospital General Docente de Calderón, Quito, ECU

**Keywords:** antibiotic-loaded bone cement, antibiotic prophylaxis, infection prevention, operative time, periprosthetic joint infection, postoperative wound drainage, preoperative optimization, primary total knee arthroplasty, staphylococcus aureus decolonization, vancomycin powder

## Abstract

Periprosthetic joint infection (PJI) remains one of the most serious complications following primary total knee arthroplasty (TKA), despite its relatively low incidence. Prevention requires addressing multiple interacting factors related to patient characteristics, microbial burden, antimicrobial prophylaxis, operative technique, and postoperative wound management.

The objective of this review is to synthesize contemporary evidence on strategies for reducing infection risk in primary TKA, with particular emphasis on clinically actionable interventions, areas of controversy, and current evidence gaps. This narrative review integrated evidence from the source manuscript and its supporting evidence base, prioritizing TKA-specific systematic reviews, meta-analyses, randomized controlled trials, registry studies, large observational cohorts, clinical guidelines, and implementation studies. Evidence was evaluated according to study design, TKA relevance, consistency of findings, susceptibility to confounding, and applicability to current clinical practice. No de novo systematic search, formal risk-of-bias assessment, or meta-analysis was performed.

Current evidence supports structured preoperative risk assessment and optimization, *Staphylococcus aureus* screening and decolonization, avoidance of intra-articular injections within three months before TKA when feasible, timely cefazolin-based systemic prophylaxis in patients without true contraindications, and stewardship-conscious antibiotic duration. Standardized antiseptic skin preparation and irrigation, efficient operative workflow, meticulous wound management, and early surveillance are also supported as components of a comprehensive prevention strategy. However, several controversies remain. Body mass index, glycemic control, nutritional status, anemia, and smoking are established risk markers, but evidence is insufficient to define universal thresholds for postponing surgery. Cefazolin remains the preferred prophylactic agent, although the optimal regimen for patients with true beta-lactam allergy or resistant-organism risk remains uncertain. Extended oral antibiotic prophylaxis may benefit selected high-risk patients but lacks sufficient evidence for routine use. Evidence regarding antibiotic-loaded bone cement and intraosseous antibiotic prophylaxis remains heterogeneous. Recent randomized evidence does not support routine intrawound vancomycin powder in primary TKA because it did not reduce infection and was associated with more minor wound complications. Persistent wound drainage also remains incompletely standardized in terms of escalation and surgical intervention.

Infection prevention in primary TKA should be approached as a risk-stratified perioperative pathway rather than as a single intervention or universal checklist. Current evidence favors systematic host optimization, microbial-burden reduction, appropriate systemic prophylaxis, standardized antisepsis, efficient high-quality surgery, and structured wound surveillance, while selective use of controversial adjuncts should be guided by patient risk and local protocols. Future research should prioritize registry-nested randomized trials, standardized definitions of high-risk patients, procedure-specific outcomes, antimicrobial-stewardship measures, cost-effectiveness, and implementation studies evaluating complete perioperative prevention bundles.

## Introduction and background

Primary total knee arthroplasty (TKA) is one of the most successful procedures in modern musculoskeletal medicine, providing substantial improvements in pain, function, and quality of life for patients with advanced knee disease. However, these benefits depend on avoiding serious postoperative complications. Among them, periprosthetic joint infection (PJI) is particularly consequential because, despite its relatively low incidence, it can result in prolonged antimicrobial treatment, repeated hospitalization, irrigation and debridement, component exchange, staged revision surgery, delayed rehabilitation, substantial healthcare costs, and considerable psychological burden. The clinical and economic consequences of infection are therefore disproportionate to its absolute frequency, making prevention a major priority in contemporary arthroplasty practice [1-3].

The problem is particularly challenging because PJI after primary TKA is multifactorial. Infection risk does not arise from a single perioperative event but from the interaction of patient-related susceptibility, microbial burden, tissue quality, surgical exposure, implant fixation, antimicrobial prophylaxis, operative workflow, and postoperative wound healing. Established risk factors include obesity, diabetes, malnutrition or hypoalbuminemia, smoking, inflammatory disease, immunosuppression, anemia, renal disease, and other comorbidities [4-8]. Several of these factors are potentially modifiable, creating an opportunity for preoperative optimization. However, the presence of an association with PJI does not necessarily establish a threshold at which surgery should be delayed or cancelled. This distinction is clinically important because rigid criteria based on isolated values such as body mass index (BMI), hemoglobin A1c (HbA1c), albumin, or smoking status may not adequately reflect an individual patient's overall risk or the potential consequences of postponing surgery [7,9-12].

In addition to host optimization, contemporary infection-prevention strategies address microbial burden and perioperative antimicrobial protection. *Staphylococcus aureus* screening and decolonization have demonstrated favorable results in elective total joint arthroplasty, although institutions continue to differ regarding universal decolonization versus screen-and-treat approaches [13-15]. Similarly, the timing of intra-articular injections before TKA remains clinically relevant. Several meta-analyses have reported an increased risk of PJI when injections are administered within three months before surgery, whereas other large database studies have not consistently reproduced this association, and emerging evidence suggests that risk may vary according to injection type and timing [16-20]. These findings illustrate a broader problem in PJI prevention: clinically important interventions may have heterogeneous evidence, making it difficult to translate individual studies into consistent perioperative recommendations.

Systemic antibiotic prophylaxis is another central component of infection prevention. Current evidence generally supports cefazolin as the preferred prophylactic agent for patients without a true contraindication, while the optimal approach for patients with beta-lactam allergy labels, resistant-organism risk, or other indications for alternative agents remains less certain [21-30]. Questions also persist regarding the duration of prophylaxis and the role of extended oral antibiotics in selected high-risk patients [21-23,31-33]. Likewise, local preventive strategies-including antibiotic-loaded bone cement, intrawound vancomycin powder, intraosseous antibiotic administration, and antiseptic irrigation-have produced inconsistent findings across randomized trials, observational studies, registries, and meta-analyses [34-46]. Operative duration and postoperative wound drainage add further complexity because both are associated with infection risk, yet the extent to which they represent modifiable causal factors versus markers of surgical complexity remains uncertain [47-50].

Despite the growing body of literature, an important clinical gap remains: the available evidence is fragmented across individual risk factors and preventive interventions, while primary TKA infection prevention is increasingly implemented as a multimodal perioperative pathway. Studies frequently evaluate one intervention at a time, use different definitions of high-risk patients, combine primary and revision arthroplasty or hip and knee procedures, and report heterogeneous infection outcomes. Consequently, findings that appear favorable in one population or setting may not be directly applicable to another. In addition, some interventions have accumulated widespread clinical use despite limited randomized evidence, whereas other potentially effective strategies remain insufficiently evaluated in risk-stratified populations. This creates uncertainty regarding which measures should be considered routine, which should be selectively applied, and which should remain investigational.

A narrative review is therefore needed to integrate these dispersed findings into a clinically coherent framework focused specifically on infection-risk optimization in primary TKA. Rather than examining individual interventions in isolation, this review evaluates the continuum of prevention from preoperative patient optimization and microbial decolonization to systemic and local antimicrobial strategies, intraoperative antisepsis and efficiency, and early postoperative wound surveillance. Particular attention is given to areas in which evidence is consistent, where studies remain contradictory, and where potential benefits must be balanced against antimicrobial resistance, adverse events, cost, and implementation burden.

The purpose of this narrative review is to synthesize current evidence on infection-risk optimization in primary TKA, identify the most clinically actionable preventive strategies, clarify areas of controversy, and define the principal evidence gaps that should guide future research. By integrating these domains into a risk-stratified perioperative framework, this review aims to help clinicians distinguish interventions supported by relatively strong evidence from those that should be individualized or reserved for selected high-risk patients, while also highlighting priorities for future randomized, registry-based, and implementation studies.

## Review

This narrative review focused on evidence addressing infection prevention and infection-related risk factors in patients undergoing primary TKA. The evidence selection prioritized studies directly evaluating primary TKA. When TKA-specific evidence was limited, high-quality total joint arthroplasty studies were considered when the intervention was biologically and operationally applicable to primary TKA and procedure-specific limitations could be acknowledged.

Inclusion criteria

Eligible evidence included systematic reviews, meta-analyses, randomized controlled trials, registry-based studies, large observational or administrative cohorts, pharmacokinetic studies directly relevant to antibiotic delivery, clinical practice guidelines, and implementation studies addressing infection prevention, perioperative risk optimization, antimicrobial prophylaxis, decolonization, local antibiotic strategies, antiseptic irrigation, operative factors, or postoperative wound management in primary TKA or applicable total joint arthroplasty populations.

Exclusion criteria

Evidence was excluded when it focused exclusively on revision arthroplasty or treatment of established PJI rather than prevention, non-arthroplasty procedures, interventions without direct relevance to primary TKA infection prevention, or evidence that could not provide clinically applicable information for the objectives of this review.

Evidence synthesis

A total of 50 references were included in the final evidence synthesis (Appendix). The evidence was evaluated according to study design, sample size, procedure specificity, consistency of findings, susceptibility to confounding, and applicability to current clinical practice. As this was a narrative review, no formal systematic-review risk-of-bias assessment, duplicate independent screening, or quantitative meta-analysis was performed. Evidence was interpreted according to study design, sample size, outcome specificity, TKA relevance, consistency across sources, susceptibility to confounding, and capacity to inform current practice. Particular attention was given to areas in which the evidence base showed agreement and contradiction, including optimization thresholds, *S. aureus* decolonization, preoperative injection timing, cefazolin versus non-cephalosporin prophylaxis, duration of systemic prophylaxis, extended oral antibiotics, antibiotic-loaded bone cement, intrawound or intra-articular vancomycin, intraosseous antibiotic delivery, antiseptic irrigation, operative time, and persistent wound drainage. The final manuscript provides a clinically integrated narrative synthesis suitable for identifying actionable concepts, controversies, and research priorities in primary TKA infection prevention.

Results

Preoperative Risk Optimization and Patient Selection

The evidence consistently identifies host optimization as a foundational component of infection prevention in primary TKA. Reviews and meta-analyses associate PJI with obesity, diabetes, malnutrition or hypoalbuminemia, smoking, inflammatory disease, immunosuppression, anemia, renal disease, cardiac disease, and postoperative glucose dysregulation [4-12]. A systematic review of preoperative risk-factor screening protocols found that structured optimization pathways were associated with reduced surgical-site infection, although the included studies were retrospective and therefore vulnerable to selection bias, secular trends, and implementation effects [8]. A recent TKA-specific meta-analysis further emphasized the cumulative nature of risk, reporting associations for prolonged operative time, obesity, hypoalbuminemia, immunosuppression, diabetes, and male sex, but it did not establish intervention thresholds or prove that modifying each factor reduces PJI in proportion to its observed association [7].

The most clinically persuasive preoperative domains are those that are both biologically plausible and modifiable. Tobacco use is supported by meta-analytic evidence showing higher wound-complication and PJI risk among current users than non-users, with former users generally showing lower risk than current users, suggesting at least partial reversibility after cessation [10]. Glycemic optimization is also relevant, but the evidence cautions against overreliance on a single HbA1c cutoff. Inadequate glycemic control is associated with surgical-site infection, yet perioperative glucose excursions may be more immediately relevant than a single preoperative measurement [11]. Malnutrition is one of the strongest and most actionable host factors; contemporary meta-analysis links malnutrition with both surgical-site infection and PJI, supporting routine nutritional screening and correction before elective TKA when feasible [12]. Overall, the evidence supports optimization protocols, but it does not support simplistic cancellation rules based on isolated thresholds without considering the broader clinical context [7-12].

Microbial Decolonization and Preoperative Injection Timing

Although *S. aureus* is an important and preventable cause of PJI, infection after primary TKA is microbiologically diverse. Coagulase-negative staphylococci and *S. aureus* are among the most frequently isolated pathogens, while streptococci, enterococci, Gram-negative bacilli, polymicrobial infections, and, less commonly, fungal organisms also contribute to the overall burden of PJI. Therefore, *S. aureus* screening and decolonization should be considered one component of a broader infection-prevention strategy rather than a comprehensive approach to all potential pathogens. A meta-analysis of elective total joint arthroplasty studies reported that nasal screening and decolonization reduced surgical-site infection, PJI, and *S. aureus* infection [13]. A preoperative hip and knee arthroplasty program using mupirocin for carriers reported favorable infection outcomes and low implementation cost, although the design was not randomized [14]. Multicenter bundle data also support standardized screening and decolonization as part of a broader approach to reducing complex *S. aureus* surgical-site infections [15]. The unresolved issue is not whether colonization matters, but whether institutions should use universal decolonization, screen-and-treat pathways, or risk-based protocols. Universal strategies may reduce missed carriers and simplify workflow, whereas selective strategies may reduce unnecessary antimicrobial exposure and resistance pressure [13-15,27,30].

Preoperative intra-articular injections create a different problem because they are common in patients awaiting TKA and because risk appears time-dependent. Meta-analyses generally suggest increased PJI risk when corticosteroid or intra-articular injections are performed within three months before primary TKA, with no clear excess risk beyond that interval [16,17]. However, large database studies have not uniformly confirmed this relationship, and drug-specific analyses suggest that risk may vary by corticosteroid type, with stronger associations reported for some agents within 90 days than for others [18-20]. The evidence therefore supports a cautious three-month avoidance interval as a pragmatic scheduling rule, but the true magnitude of risk, the relative contribution of corticosteroid versus hyaluronic acid injections, and drug-specific effects remain incompletely resolved [16-20].

Systemic Antibiotic Prophylaxis and Duration

Systemic antibiotic prophylaxis remains the most evidence-based intraoperative intervention. Foundational guideline and systematic-review evidence support timely perioperative antibiotics and discontinuation within a stewardship-conscious window for routine cases [21-23]. Contemporary evidence supports cefazolin as the preferred prophylactic backbone in patients without true contraindications [21,23-26]. In a cohort of 11,550 primary TKAs performed with a background prevention protocol, non-cephalosporin prophylaxis with vancomycin or clindamycin was associated with a higher 90-day PJI rate than cefazolin [24]. A large Norwegian register-based study of primary hip, knee, and hemiarthroplasties found no advantage to additional prophylactic doses and reported the lowest PJI risk with cefazolin-containing regimens [26]. These data support preserving cefazolin as the default prophylactic agent and encourage careful allergy assessment or delabeling when appropriate [21,23-26].

The controversy lies in patients labeled as beta-lactam allergic or colonized with resistant organisms. A Norwegian registry study of cemented primary total knee replacements found no difference in revision for infection between clindamycin and cephalosporin prophylaxis, directly contrasting with institutional studies reporting worse outcomes with non-cephalosporin regimens [24,25]. Differences in endpoints, confounding by indication, cement use, vancomycin dosing, local microbiology, and allergy-label accuracy likely explain part of this discrepancy. Dual prophylaxis also remains selective rather than routine because data have not consistently shown broad benefit and because vancomycin addition may increase nephrotoxicity in some cohorts [28-30]. Thus, antimicrobial decisions should combine accurate allergy history, methicillin-resistant *S. aureus* (MRSA)/methicillin-sensitive *S. aureus* (MSSA) status, weight-adjusted dosing, correct timing, and local resistance patterns rather than default escalation [21,24-30].

Duration is similarly unsettled. Systematic reviews and guideline-based practice generally do not support routine antibiotic continuation beyond 24 hours for unselected primary arthroplasty [21-23]. Meta-analytic evidence comparing single-dose, perioperative, and extended regimens does not provide a consistent TKA-specific justification for routine prolonged prophylaxis [23,31,32]. Conversely, a large single-center high-risk cohort found that seven days of oral antibiotics after discharge was associated with lower one-year PJI in high-risk total joint arthroplasty patients [33]. This finding is clinically important but remains vulnerable to confounding by protocolized care, patient selection, concurrent interventions, and local microbiology. Extended oral antibiotics therefore remain a high-interest but insufficiently proven strategy for routine primary TKA and should be restricted to carefully governed high-risk protocols or research settings that track adverse events, resistance, and *Clostridioides difficile* infection [21-23,31-33].

Local Antibiotic Strategies and Antiseptic Irrigation

Antibiotic-loaded bone cement is one of the most persistent controversies in primary TKA. Early randomized and comparative studies suggested possible benefit in selected populations, particularly in patients with diabetes, and contemporary meta-analysis by Xu et al. suggested that antibiotic-impregnated cement may reduce deep infection in primary TKA [34-36]. However, another large meta-analysis found no significant PJI reduction with antibiotic-loaded cement compared with plain cement in primary TKA and raised concerns about adverse events, cost, and routine use [37]. Registry analyses are also mixed. The UK National Joint Registry reported lower revision risk and a trend toward fewer infection revisions with antibiotic-loaded cement, whereas Kaiser Permanente data suggested that commercially prepared antibiotic-loaded cement did not confer a clear overall infection advantage in unselected cemented primary TKA, with possible benefit limited to some higher-risk subgroups [38,39]. Economic analyses further emphasize that universal use must be justified by absolute risk reduction, cost, and local infection rates rather than biological plausibility alone [40]. These contradictions suggest that routine use in all primary TKAs is not conclusively justified, but selective use in high-risk patients remains plausible [34-40].

Local antibiotic delivery excluding cement is also unsettled. Earlier systematic reviews of intrawound vancomycin and other local antibiotics suggested possible reduction in deep infection, but these benefits were derived largely from observational studies and were accompanied by concerns about wound complications, heterogeneous dosing, and resistance [42,43]. The most important recent evidence is a prospective double-blind randomized trial of 1,022 primary TKA patients showing no reduction in PJI or surgical-site infection with intrawound vancomycin powder and significantly more minor wound complications, especially among patients with diabetes [41]. This trial weakens the rationale for routine intrawound vancomycin in primary TKA. Intraosseous regional antibiotic prophylaxis is promising; meta-analysis suggested lower surgical-site infection and PJI compared with intravenous prophylaxis, but the small number of studies and technique heterogeneity limit adoption outside selected protocols or research settings [44].

Antiseptic irrigation appears more favorable because of low cost, broad-spectrum plausibility, and lower systemic antimicrobial pressure. Dilute povidone-iodine lavage has been associated with reduced early deep infection in arthroplasty cohorts, and a recent meta-analysis found that both chlorhexidine and povidone-iodine irrigation reduced PJI compared with saline in total joint arthroplasty, without clear superiority between antiseptic agents [45,46]. Nevertheless, concentration, exposure time, lavage volume, mechanical technique, timing relative to implantation, and co-interventions vary substantially across studies [45,46]. Standardization of irrigation protocols is therefore essential before comparing outcomes between institutions.

Operative Time and Surgical Efficiency

Operative time is consistently associated with infection. A meta-analysis of 427,361 primary TKAs found higher PJI risk in procedures exceeding common duration thresholds, and machine-learning work identified prolonged operative time as an important perioperative predictor [47,48]. Whether time is causal or a marker of complexity, efficient workflow should be treated as an infection-prevention objective. The practical goal is controlled efficiency rather than speed alone: preoperative planning, experienced assistance, instrumentation readiness, minimizing unnecessary room traffic, decisive exposure, meticulous hemostasis, and high-quality closure [47,48,50].

Postoperative Wound Management and Persistent Drainage

Early postoperative wound problems are clinically important because persistent drainage, superficial complications, and delayed healing can create a pathway to deep infection. The evidence base is less robust than for antibiotic prophylaxis or major host risk factors, but it supports structured surveillance and rapid escalation when drainage persists [49,50]. Protocol-based work suggests that time-dependent management of persistent drainage may reduce PJI, but optimal thresholds for dressing changes, aspiration, negative-pressure therapy, antibiotic avoidance, anticoagulation adjustment, and return to the operating room remain incompletely standardized [49].

Integrated High-Risk Prevention Pathways

High-risk pathways should integrate rather than simply accumulate interventions. Patients with obesity, diabetes, malnutrition, tobacco exposure, inflammatory disease, immunosuppression, anemia, renal disease, recent injections, *S. aureus* colonization, or complex operative requirements may benefit from intensified screening, multidisciplinary optimization, strict perioperative glucose control, decolonization, cefazolin allergy evaluation, selective cement or local strategies, antiseptic irrigation, experienced surgical teams, and closer wound follow-up [4-15,31-50]. However, the evidence does not support adding every possible intervention to every high-risk patient. The challenge is to distinguish additive benefit from redundant or harmful escalation, particularly when interventions such as extended antibiotics and local vancomycin introduce antimicrobial-stewardship and wound-healing risks [21-23,31-33,41-43].

Discussion

Preoperative Risk Optimization and Patient Selection

The available evidence consistently identifies obesity, diabetes, smoking, malnutrition or hypoalbuminemia, immunosuppression, anemia, renal disease, and inflammatory conditions as important risk factors for PJI following primary TKA [4-12]. Structured preoperative screening and optimization programs are associated with lower infection rates; however, most supporting studies are observational and do not establish that modifying an individual risk factor directly reduces PJI. In particular, the evidence does not support universal postponement thresholds based solely on BMI, HbA1c, albumin, or smoking status. These variables should therefore be interpreted within the patient's overall comorbidity burden, nutritional status, soft-tissue condition, and surgical complexity [7-12].

S. aureus Decolonization and Preoperative Injections

Screening and decolonization for *S. aureus* have demonstrated reductions in surgical-site infection, PJI, and *S. aureus*-specific infection in elective arthroplasty populations [13-15]. However, because PJI is caused by multiple organisms, decolonization should be considered one component of a broader infection-prevention strategy. Regarding intra-articular injections, systematic reviews and large observational studies generally indicate an increased risk of PJI when corticosteroid or other intra-articular injections are performed within three months before TKA [16-20]. Nevertheless, inconsistent findings across studies and differences according to injection type suggest that the magnitude of this risk remains uncertain. A three-month avoidance interval therefore represents a pragmatic risk-reduction strategy rather than an absolute evidence-based threshold.

Systemic Antibiotic Prophylaxis

Systemic antibiotic prophylaxis represents one of the most consistently supported interventions. Cefazolin is generally associated with favorable outcomes and remains the preferred prophylactic agent for patients without a true contraindication [21-26]. Evidence comparing cefazolin with clindamycin is not entirely consistent, particularly across registry and institutional studies, highlighting the potential influence of allergy labeling, local microbiology, and confounding by indication [24-26]. Accurate allergy assessment, appropriate weight-based dosing, correct timing, and consideration of MRSA status are therefore important components of prophylaxis [21,24-30]. Routine dual prophylaxis has not demonstrated sufficiently consistent benefit and may increase adverse events, including acute kidney injury [28-30].

Duration of Antibiotic Prophylaxis

Guidelines and systematic reviews generally support discontinuation of prophylactic antibiotics within 24 hours for routine primary arthroplasty [21-23,31,32]. Extended oral antibiotic prophylaxis has shown promising results in selected high-risk populations, including a large cohort in which seven days of postoperative oral antibiotics was associated with lower one-year PJI rates [33]. However, the observational nature of this evidence and potential confounding from concurrent prevention measures limit its generalizability. Consequently, extended prophylaxis cannot currently be recommended as a routine strategy for all primary TKA patients and should remain limited to carefully defined high-risk protocols or research settings [31-33].

Local Antibiotic Strategies and Antiseptic Irrigation

Evidence regarding antibiotic-loaded bone cement remains conflicting. Some randomized studies, meta-analyses, and registry analyses suggest a potential reduction in infection or revision, particularly in selected high-risk patients, whereas other large studies have not demonstrated a significant overall benefit in unselected primary TKA [34-40]. These inconsistencies are influenced by differences in cement formulation, antibiotic dose, fixation method, patient risk, and outcome definitions. Current evidence therefore supports selective rather than universal use.

Intrawound vancomycin has similarly produced inconsistent findings in observational studies. Importantly, a recent prospective randomized trial involving 1,022 primary TKA patients found no reduction in PJI or surgical-site infection and reported more minor wound complications with vancomycin powder [41]. This finding provides stronger evidence against routine use in primary TKA. Intraosseous antibiotic prophylaxis remains promising, but the available evidence is limited by the small number of studies and methodological heterogeneity [42-44].

Antiseptic irrigation appears to have a more favorable evidence profile. Studies and meta-analyses suggest that dilute povidone-iodine or chlorhexidine irrigation may reduce postoperative infection compared with saline [45,46]. However, differences in concentration, exposure time, irrigation volume, and operative technique prevent identification of a clearly superior protocol.

Operative Time and Postoperative Wound Management

Prolonged operative time is consistently associated with increased PJI risk in primary TKA [47,48]. However, operative duration may also reflect patient complexity, difficult exposure, obesity, deformity, and surgical team experience. The evidence therefore supports efficient and standardized operative workflow rather than simply pursuing shorter surgical times.

Persistent postoperative wound drainage represents another clinically relevant risk factor for subsequent infection [49,50]. Available evidence supports early recognition and structured management, but substantial variation remains regarding anticoagulation modification, aspiration, negative-pressure therapy, antibiotic administration, and timing of surgical intervention. Thus, wound management remains an important area in which standardized, evidence-based algorithms are needed.

Overall Evidence Synthesis

Overall, the evidence supports a multimodal, risk-stratified approach to infection prevention in primary TKA. The most consistent evidence favors structured preoperative optimization, *S. aureus* decolonization, avoidance of intra-articular injections within three months when feasible, cefazolin-based systemic prophylaxis, standardized antisepsis, efficient operative workflow, and early wound surveillance [4-15,21-23,45-50]. In contrast, evidence remains heterogeneous or insufficient to support universal use of extended oral antibiotics, antibiotic-loaded bone cement, intraosseous antibiotics, or intrawound vancomycin. These findings indicate that prevention should focus on interventions with established benefit while reserving uncertain adjunctive strategies for selected high-risk patients or appropriately designed clinical studies.

Clinical implications

In current practice, infection-risk optimization before primary TKA should begin with a structured, documented pathway. Patients should be screened for diabetes and perioperative glucose risk, nutritional deficiency or hypoalbuminemia, tobacco use, severe obesity or adverse soft-tissue envelope, anemia, renal or cardiac disease, inflammatory arthritis, immunosuppression, prior injections, and *S. aureus* colonization [4-20]. Modifiable factors should be treated before surgery whenever doing so is feasible and clinically meaningful. Surgical timing should be individualized, but injections within three months before TKA should generally be avoided unless there is a compelling reason to proceed and the patient accepts the uncertain residual risk [16-20].

For antimicrobial prophylaxis, cefazolin should remain the default agent in patients without true contraindications [21,23-26]. Institutions should invest in allergy clarification because inaccurate beta-lactam allergy labels may push patients toward less effective or more complex regimens [24-30]. Vancomycin should be reserved for appropriate indications such as MRSA colonization or true severe beta-lactam allergy and should be correctly weight-dosed and timed [21,27-30]. Routine extension of antibiotics beyond 24 hours is not supported for unselected primary TKA [21-23,31,32]. Extended oral antibiotics may be considered only within locally approved high-risk protocols that track adverse events, resistance, and infection outcomes [31-33].

For intraoperative adjuncts, antiseptic skin preparation and standardized antiseptic irrigation are reasonable low-risk components of a prevention bundle, provided concentrations and exposure times are protocolized [21,22,27,30,45,46]. Antibiotic-loaded bone cement may be considered selectively for high-risk patients, but routine universal use remains controversial [34-40]. Intrawound vancomycin powder should not be used routinely in primary TKA based on current randomized evidence showing no infection reduction and increased minor wound complications [41]. Operative efficiency should be improved through planning, team consistency, and workflow design, not by compromising surgical quality [47,48,50]. Persistent wound drainage should trigger a predefined escalation algorithm rather than ad hoc antibiotic prescriptions or delayed recognition [49,50].

Limitations

This narrative review has several limitations. First, the evidence synthesis was specifically focused on primary TKA. Studies exclusively addressing revision arthroplasty, total hip arthroplasty, or other procedures were excluded. When studies included mixed arthroplasty populations, only findings specifically reported for primary TKA were considered in the evidence synthesis.

Second, the available literature is heterogeneous in study design, patient characteristics, definitions of high-risk status, infection outcomes, and preventive protocols. Evidence for several interventions is based predominantly on observational studies, registries, or meta-analyses of heterogeneous studies, which may be affected by confounding, selection bias, and differences in institutional prevention protocols. Randomized evidence is available for some interventions, but important preventive strategies remain insufficiently evaluated in primary TKA.

Finally, because this was a narrative review rather than a de novo systematic review, no formal duplicate screening, standardized risk-of-bias assessment, or quantitative meta-analysis was performed. These limitations should be considered when interpreting the strength of the available evidence and the clinical implications presented in this review.

## Conclusions

Infection prevention in primary TKA is best approached as a multimodal, risk-stratified perioperative strategy rather than a single intervention. The available evidence most consistently supports structured preoperative identification and optimization of modifiable risk factors, *S. aureus* screening and decolonization, avoidance of intra-articular injections within three months before surgery when feasible, cefazolin-based perioperative prophylaxis in patients without true contraindications, standardized antiseptic practices, efficient surgical workflow, and early recognition and management of postoperative wound problems. Several commonly used preventive strategies remain uncertain. Universal antibiotic-loaded bone cement and extended postoperative oral antibiotics are not sufficiently supported for routine use in all primary TKA patients, although selective use in carefully defined high-risk populations may be reasonable. Current randomized evidence does not support routine intrawound vancomycin powder because it has not demonstrated a reduction in infection and may increase minor wound complications. Evidence for intraosseous antibiotic prophylaxis and specific wound-management protocols remains limited or heterogeneous.

Overall, the evidence favors individualized prevention based on patient risk and the strength of available evidence, while avoiding unnecessary antimicrobial escalation. Future research should prioritize primary TKA-specific, risk-stratified randomized and implementation studies that evaluate complete prevention pathways rather than isolated interventions, while incorporating antimicrobial resistance, adverse events, cost-effectiveness, and patient-centered outcomes.

## Appendices

**Table 1:** Studies Supporting the Rationale and Evidence Base for Periprosthetic Joint Infection (PJI) Prevention in Total Knee Arthroplasty (TKA) THA: total hip arthroplasty; SSI: surgical-site infection

Reference	Study	Year	Study type	Topic/risk factor or intervention	Evidence used to justify the study
[1]	Kurtz et al.	2012	Economic/epidemiologic study	Economic burden of PJI	Demonstrates the substantial economic impact of PJI and supports the importance of prevention.
[2]	Kurtz et al.	2010	Epidemiologic study	Infection burden after hip and knee arthroplasty	Establishes the clinical burden of infection following joint arthroplasty.
[3]	Tande and Patel	2014	Review	Prosthetic joint infection	Provides an overview of epidemiology, pathogenesis, diagnosis, and management of PJI.
[4]	George et al.	2017	Review	Risk factors for lower-limb PJI	Supports the concept that PJI is associated with multiple patient- and procedure-related risk factors.
[5]	Rodriguez-Merchan and Delgado-Martinez	2022	Review	Risk factors after primary TKA	Specifically summarizes risk factors associated with PJI following primary TKA.
[6]	Kunutsor et al.	2016	Systematic review/meta-analysis	Patient-related risk factors	Provides pooled evidence that several patient characteristics are associated with increased PJI risk.
[7]	Li et al.	2025	Systematic review/meta-analysis	Risk factors after primary TKA	Provides updated evidence regarding risk factors specifically after primary TKA.
[8]	Johns et al.	2020	Systematic review	Preoperative screening	Supports systematic identification and optimization of modifiable risk factors before arthroplasty.
[9]	Chan et al.	2020	Review	Preoperative optimization	Supports optimization of high-risk patients before TKA/THA to reduce PJI risk.
[10]	Bedard et al.	2019	Systematic review/meta-analysis	Tobacco use	Supports smoking/tobacco use as an important modifiable risk factor for wound complications and PJI.
[11]	Shohat et al.	2018	Systematic review/meta-analysis	Glycemic control	Supports the association between inadequate glycemic control and increased SSI risk.
[12]	Chen and Chen	2024	Systematic review/meta-analysis	Malnutrition	Supports malnutrition as a risk factor for PJI and SSI after joint arthroplasty.
[13]	Zhu et al.	2020	Systematic review/meta-analysis	*Staphylococcus aureus* screening/decolonization	Evaluates whether nasal screening and decolonization can reduce SSI and prosthesis-related infection.
[14]	Yuste Berenguer et al.	2023	Clinical study	*S. aureus* screening/decolonization	Provides clinical evidence regarding implementation of a preoperative *S. aureus* screening/decolonization program.
[15]	Schweizer et al.	2015	Intervention study	Infection-prevention bundle	Supports use of bundled infection-prevention interventions to reduce SSI.
[16]	Avila et al.	2022	Cohort/observational study	Intra-articular injection ≤ 3 months before TKA	Supports an association between recent intra-articular injection and increased PJI risk.
[17]	Kim et al.	2023	Systematic review/meta-analysis	Preoperative corticosteroid injection	Supports increased PJI risk when steroid injection is performed within 3 months before TKA.
[18]	Kurtz et al.	2022	Study/review	Corticosteroid or hyaluronic acid injections	Provides contrasting evidence suggesting that intra-articular corticosteroid/hyaluronic acid injections may not universally increase PJI risk.
[19]	Muffly et al.	2024	Clinical study	Timing/type of corticosteroid injections	Evaluates whether different corticosteroid injections carry different PJI risks.
[20]	Nin et al.	2025	Clinical study	Joint injection/aspiration before TKA	Provides recent evidence concerning aspiration or injection before TKA and subsequent PJI risk.
[21]	Bratzler et al.	2013	Clinical practice guideline	Antimicrobial prophylaxis	Establishes evidence-based principles for antimicrobial prophylaxis in surgery.
[22]	Berríos-Torres et al.	2017	Clinical practice guideline	SSI prevention	Provides guideline recommendations for prevention of SSI.
[23]	Siddiqi et al.	2019	Systematic review/meta-analysis	Perioperative antibiotic prophylaxis	Evaluates evidence supporting perioperative antibiotic prophylaxis in total joint arthroplasty.
[24]	Buchalter et al.	2022	Clinical study	Cefazolin prophylaxis	Supports cefazolin as a central antibiotic for prevention of acute PJI after primary TKA.
[25]	Pawloy et al.	2023	Registry study	Clindamycin vs cephalosporins	Reports comparative infection/revision risk according to antibiotic prophylaxis regimen.
[26]	Lutro et al.	2025	Registry-based study	Number/type of antibiotic doses	Evaluates optimal systemic antibiotic prophylaxis regimens using a very large arthroplasty registry.
[27]	WHO	2016	Global guideline	SSI prevention	Provides international recommendations for prevention of SSI.
[28]	Sewick et al.	2012	Comparative clinical study	Dual antibiotic prophylaxis	Evaluates whether adding a second antibiotic improves SSI prevention after joint arthroplasty.
[29]	Courtney et al.	2015	Cohort study	Vancomycin + cefazolin	Highlights potential harms of dual prophylaxis, particularly acute kidney injury.
[30]	Allegranzi et al.	2016	Evidence-based guideline/review	Preoperative SSI prevention	Supports evidence-based preoperative measures for reducing SSI.
[31]	Dasari et al.	2024	Meta-analysis	Extended antibiotic prophylaxis	Evaluates whether extended antibiotics reduce infection after primary and aseptic revision arthroplasty.
[32]	Fernandes et al.	2024	Systematic review/meta-analysis	Single-dose vs extended prophylaxis	Directly evaluates duration of antibiotic prophylaxis in primary hip and knee arthroplasty.
[33]	Kheir et al.	2021	Large cohort study	Extended oral antibiotics	Reports outcomes of extended oral antibiotic prophylaxis in high-risk arthroplasty patients.
[34]	Chiu et al.	2001	Prospective randomized study	Antibiotic-loaded cement	Evaluates cefuroxime-impregnated cement in diabetic patients undergoing primary TKA.
[35]	Jiranek et al.	2006	Review	Antibiotic-loaded bone cement	Reviews the rationale and evidence for antibiotic-loaded cement as infection prophylaxis.
[36]	Xu et al.	2022	Meta-analysis	Antibiotic-loaded vs plain cement	Evaluates whether antibiotic-loaded cement reduces PJI compared with plain cement in primary TKA.
[37]	Li et al.	2022	Systematic review/meta-analysis	Antibiotic-loaded cement	Provides pooled evidence regarding effectiveness of antibiotic-loaded cement in primary TKA.
[38]	Jameson et al.	2019	Registry study	Antibiotic-loaded cement	Very large registry analysis evaluating revision risk associated with antibiotic-loaded cement.
[39]	Namba et al.	2020	Large observational study	Commercial antibiotic-loaded cement	Evaluates infection risk after cemented primary TKA using commercially prepared antibiotic-loaded cement.
[40]	Yayac et al.	2019	Economic/clinical study	Antibiotic cement	Evaluates cost and infection outcomes, questioning whether antibiotic cement provides sufficient infection reduction to justify its additional cost.
[41]	Mulpur et al.	2024	Prospective randomized controlled trial	Intrawound vancomycin	Directly evaluates efficacy of local vancomycin in preventing PJI after primary TKA.
[42]	Movassaghi et al.	2022	Systematic review/meta-analysis	Intrawound vancomycin	Evaluates available evidence for topical vancomycin and emphasizes the need for high-quality randomized trials.
[43]	Saidahmed et al.	2021	Systematic review/meta-analysis	Local antibiotics	Evaluates local antibiotic strategies in primary hip and knee arthroplasty.
[44]	Lei et al.	2023	Systematic review/meta-analysis	Intraosseous antibiotics	Evaluates regional intraosseous antibiotic prophylaxis compared with intravenous administration in TKA.
[45]	Brown et al.	2012	Clinical study	Dilute povidone-iodine lavage	Supports dilute betadine lavage before wound closure as an infection-prevention strategy.
[46]	Machinski et al.	2025	Systematic review/meta-analysis	Chlorhexidine/povidone-iodine vs saline irrigation	Provides recent pooled evidence regarding antiseptic irrigation solutions during primary arthroplasty.
[47]	Shin et al.	2024	Meta-analysis	Operative time	Supports prolonged operative time as a risk factor for PJI after primary TKA.
[48]	Chong et al.	2025	Machine-learning study	Prediction of PJI	Demonstrates the potential to integrate preoperative and perioperative variables to predict PJI risk.
[49]	Leggieri et al.	2026	Clinical study	Persistent wound drainage	Evaluates a time-dependent wound-management protocol intended to prevent PJI in patients with persistent drainage.
[50]	Ratto et al.	2016	Review	Comprehensive PJI prevention	Summarizes practical surgical strategies that can be implemented to reduce infection risk after TKA.
